# The Potential Roles of Myokines in Adipose Tissue Metabolism with Exercise and Cold Exposure

**DOI:** 10.3390/ijms231911523

**Published:** 2022-09-29

**Authors:** Shu Jiang, Jun-Hyun Bae, Yangwenjie Wang, Wook Song

**Affiliations:** 1 Health and Exercise Science Laboratory, Institute of Sports Science, Department of Physical Education, Seoul National University, Seoul 08826, Korea; 2 Institute of Sports & Arts Covergence, Inha University, Incheon 22212, Korea; 3 School of Sport Science, Beijing Sport University, Beijing 100084, China; 4 Institute on Aging, Seoul National University, Seoul 08826, Korea

**Keywords:** myokine, skeletal muscle, adipose tissue, exercise, cold exposure

## Abstract

Exercise and cold exposure are two stimuli that have been suggested as solely effective to modulate adipose tissue metabolism and improve metabolic health in obese populations. The two primary organs involved in energy metabolism during exercise and/or cold exposure are skeletal muscle and adipose tissue. Adipose tissue can be divided mainly into two types: white adipose tissue (WAT), which primarily stores energy, and brown adipose tissue (BAT), known as the primary source of thermogenesis. The exercise-stimulated release of myokines allows for crosstalk between skeletal muscle and adipose tissue, partially mediating the beneficial effects of exercise. Cold exposure is another trigger for the regulation of myokine secretions, thus increasing adipose tissue metabolism, especially via activation of BAT. Therefore, this has generated the hypothesis that exercise in conjunction with cold exposure might be the optimal regimen to regulate myokine profiles and gain more beneficial health effects. However, to date, human experimental data regarding different exercise (frequency, type, time and intensity) and cold exposure (temperature, time and frequency) patterns are scarce. In this review, we will summarize the current human clinical trials investigating the regulation of myokines induced by exercise combined with cold exposure, to elaborate on the roles of myokines in mediating adipose tissue metabolism.

## 1. Introduction

Although obesity has been studied for several decades, the overall prevalence has still increased dramatically and has affected a large number of people all over the world. Obesity is a strong risk factor for several chronic diseases, including type 2 diabetes, insulin resistance, hypertension, sarcopenia and some types of cancer [1]. It has been widely accepted that exercise and physical activity are potent therapies against the progression of these chronic diseases. However, the effect of exercise is rarely enough, and intensive exercise regimens would not be practical for some particular populations, for example, older people and those with some diseases. Thus, novel strategies are necessary to prevent and treat obesity and boost energy expenditure.

Most exercise intervention studies were carried out in a neutral thermal environment (20–25 °C), but temperatures higher or lower than neutral temperature can have an impact on exercise performance and energy metabolism. Due to global climate change, negative health effects caused by hot ambient conditions have been widely studied [2]. On the other hand, potential health benefits after cold exposure have gained considerable interest recently.

A few studies have demonstrated the powerful capability of cold exposure or cold acclimation to improve the metabolic profile. For example, daily cold exposure increased the brown adipose tissue (BAT) volume (mL) and oxidative capacity in humans [3]. In addition, regular ice/winter swimming seemed to have a positive effect on the endocrine system, decreasing triglycerides and increasing insulin sensitivity [4,5]. Recently, Munten et al. demonstrated that high-intensity interval training (HIIT) in the cold (0 °C) resulted in higher lipid oxidation rates compared with a thermoneutral environment [6]. These findings have generated the hypothesis that more beneficial effects can be achieved via exercising combined with low temperature.

**Figure 1:**
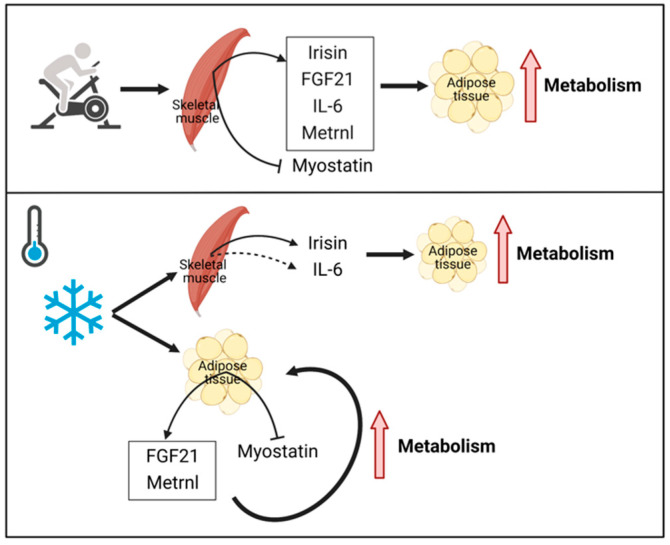
Myokines involved in adipose tissue metabolism with exercise and cold exposure. In humans, myokines (irisin, FGF21, IL-6, Metrnl and myostatin) secreted by skeletal muscle in response to exercise play important roles in adipose tissue metabolism. On the other hand, cold exposure can induce the expression of irisin in skeletal muscle, FGF21 and Metrnl in adipose tissue, and inhibit the expression of myostatin in adipose tissue, thus increasing energy metabolism. Abbreviations: FGF21: fibroblast growth factor 21; IL-6: interleukin 6; Metrnl: Meteorin-like.

Both exercise and cold exposure can induce the secretion of some circulating factors, which play roles in altering metabolic homeostasis and insulin resistance [7,8]. So, these factors might be novel therapeutic targets for metabolic diseases, including obesity and type 2 diabetes mellitus. Among these factors, proteins secreted from contracting skeletal muscle working in a paracrine, autocrine and endocrine way are factors termed “myokines”. This term was first introduced by Bengt Saltin in 2003 [9]. Myokines play a critical role in the communication between skeletal muscle and other organs such as adipose tissue, liver, brain and pancreas, and this can account partially for the beneficial effects of exercise. Previous researchers have revealed that several myokines participate in the crosstalk between skeletal muscle and adipose, including irisin, fibroblast growth factor 21 (FGF21), interleukin 6 (IL-6), Meteorin-like (Metrnl) and myostatin [7,8,10,11] (Figure 1). As a result of this crosstalk, fat metabolism and insulin sensitivity are altered. Key effects exist in exercise duration and intensity. 

Interestingly, cold exposure has also been reported to facilitate modulating the expression and release of myokines, thus increasing adipose tissue metabolism [12]. In this context, it was hypothesized that exercise combined with cold exposure might be the optimal regimen to regulate the myokine profile and a couple of studies have been conducted. However, the range of changes in myokines in response to exercise coupled with cold exposure still remains an open question. Although exercise’s sole influence on myokines has been well documented, the understanding of human metabolic responses to exercise in conjunction with cold exposure is still limited, and data regarding different exercise and cold exposure patterns are scarce. Therefore, the purpose of this review is to summarize the available clinical human trials investigating the regulation of myokines induced by the combination of exercise and cold exposure to see if exercise in the cold has an additive beneficial effect on humans. Systematic literature searches were conducted on the impact of exercise combined with cold exposure on myokine secretion in humans. Due to the limited literature on myokine induction from exercising in the cold, the systematic search of the literature was not limited to a specific myokine.

## 2. Similarities between Exercise- and Cold-induced Adaptations in Terms of Adipose Tissue Metabolism

Similarities in the health benefits of exercise and cold exposure are mainly related to adipose tissue metabolism. Mammalian adipose tissue can be divided into two types: white adipose tissue (WAT) and BAT. WAT primarily stores energy as triglycerides, while BAT is known to dissipate energy by upregulation of the expression of mitochondrial uncoupling protein 1 (UCP1), a BAT-specific activation marker [13]. UCP1 uncouples mitochondrial oxidative phosphorylation, dissipating energy as heat instead of ATP production, and, therefore, plays an important role in the regulation of body weight in humans and rodents [14]. Peroxisome proliferator-activated receptor gamma (PPARγ) coactivator-1 alpha (PGC-1α) is the key regulator of UCP1-mediated thermogenesis in BAT, which is sensitive to temperature [15,16].

In addition to its effects on skeletal muscle and the cardiovascular system, exercise was recently demonstrated to result in adaptations of adipose tissue, and, therefore, improve whole-body metabolic health. Conflicting results exist regarding the effects of exercise on BAT between human and animal studies [17]. However, adaptations of WAT were more widely researched. Exercise reduced WAT adipocyte size and lipid content, increased mitochondrial activity and induced browning of WAT [18]. 

Additionally, cold exposure was reported to increase beta-adrenergic and/or UCP1 activity, thereby enhancing thermogenesis and fat metabolism in BAT [19]. The fact that both exercise and cold exposure can promote the conversion of BAT-like phenotype among white adipocytes, a process referred to as “browning of WAT”, mainly in rodents, has suggested the potential of the two stimuli in mediating energy expenditure [17,20]. To explain this phenomenon, several hypotheses have been proposed, for instance, increased sympathetic innervation [21]. Furthermore, studies have shown that several myokines released by contracting skeletal muscles in the cold are correlated with WAT browning (Figure 1). Detailed roles of these myokines will be discussed in the following sections.

Other than browning, both exercise and cold are involved in the regulation of lipid metabolism. Although the effects of exercise on BAT metabolism have not been studied thoroughly, WAT lipolysis increases significantly after exercise in both rodents and humans [22,23]. Cyclic adenosine monophosphate (cAMP), which determines the rate of lipolysis in adipose tissue, increases in response to low temperature [24], indicating that cold exposure also increases the rate of lipid metabolism. Exercise- and cold-induced factors play roles in lipid metabolism. For example, both rodent and human studies have demonstrated that FGF21 can enhance lipolysis and fat oxidation. FGF21KO mice exhibited greater lipid stores in BAT [25]. Due to the similarities between adaptations induced by exercise and cold exposure, the combination of the two stimuli was regarded as a potential strategy for eliciting more health benefits.

## 3. Key Myokines Involved in Adipose Tissue Metabolism with Exercise and Cold Exposure

The therapeutic potential of myokines in metabolic diseases is evident. By linking skeletal muscle to other organs (adipose tissue, liver, pancreas, brain, etc.), myokines can explain positive alterations in response to stimuli. The expression and release of myokines are affected by both exercise and environmental temperature, thus mediating adipose tissue metabolism. An overview of the exercise- and cold-regulated myokines to be discussed is given in Table 1.

### 3.1. Irisin

Irisin, a contraction-regulated myokine, was first identified in 2012 as the secreted form of fibronectin type III domain containing 5 (FNDC5) mediated by the transcriptional coactivator PGC-1α [26]. As a novel myokine, irisin has gained much attention recently due to its therapeutic potential in obesity and diabetes mellitus. Enhanced irisin levels tend to facilitate energy expenditure by upregulating UCP1 levels, and thus inducing WAT browning [26]. Further, previous studies have shown that irisin plays roles in glucose/lipid homeostasis, bone metabolism and the central nervous system [36,37].

Although conflicting results still exist, both animal and human studies have shown that circulating levels of irisin were affected by exercise and low temperature [26,27,38]. Cold-induced thermogenesis includes nonshivering thermogenesis (NST) and shivering thermogenesis (ST). ST linked cold to exercise-induced thermogenesis and is associated with irisin secretion [27].

Taken together, it is possible to increase irisin secretion and optimize the exercise effects by combining exercise and cold exposure. A study conducted by Ulupinar et al. in 2021 showed that aerobic running exercise (40 min, 70% HR_max_) at 0 °C resulted in a more significant serum irisin increase [39] than running at 12 °C and 24 °C. Although the increase was not statistically significant (*p* = 0.06), upregulation of serum irisin was also observed after the Yukon Arctic Ultra, the longest and coldest ultra in the world, at −25–−2 °C [40]. The limited sample size (n = 8) might have impacted the result. Nevertheless, 40 min of running at −5–5 °C for 18 weeks (65–70% HR_max_, 4 days/week) and 60 min of cycling at 15–19 °C (60% HR_max_) and at 7 °C (60% W_max_) all showed no change in plasma irisin concentration [41,42,43]. In these studies, the temperature conditions were not low enough to elicit shivering-related muscle contraction, which might explain the lack of increase in the irisin concentration [27]. As for a shorter duration of cold exposure, 3-min whole-body cryostimulation (WBC) followed by 60 min of HIIT at 90% HR_max_ (3 times/week) [44] and 50 min of resistance training at 70–80% 1RM (3 times/week) [45] did not change irisin concentration after exercise (Table 2). Therefore, besides exercise intensity and duration, other effects on irisin levels might also include temperature and duration of cold exposure. In addition, all the aforementioned studies used ELISA kits to detect circulating irisin levels. It was reported that measurements of plasma irisin levels were precarious due to a specificity problem in the commercial ELISA kits. Hence, further research is needed to investigate whether mass spectrometry techniques are more sensitive and accurate for detecting circulating irisin, and this may alter some of the research results [46].

Furthermore, it was reported that exercise-induced irisin secretion seemed to be accentuated in older adults and increased in response to cold exposure in obese subjects [38]. However, to date, no older population is included in the experiments involving exercise in conjunction with cold exposure. Therefore, further studies need to be conducted considering age- and body composition-related differences.

### 3.2. FGF21

FGF21, released mainly by hepatocytes [47], can also be induced in skeletal muscle through the phosphatidylinositol 3-kinase (PI3-kinase)/Akt1 pathway [28]. As a member of the fibroblast growth factor superfamily, FGF21 is involved in lipid and glucose metabolism in skeletal muscle and adipose tissue. 

The FGF21 response to exercise is still ambiguous. Despite inconsistent results following exercise [48,49,50], circulating FGF21 levels tend to increase in humans, especially after acute exercise [49,51]. On the other hand, both rodent and human studies showed that prolonged low temperatures induced the expression of FGF21 in adipose tissue, thereby enhancing lipolysis and thermogenesis responses through WAT browning [27,29]. Therefore, like irisin, increased FGF21 expression benefits energy metabolism. However, unlike irisin, which is mainly induced by muscle contraction when ST occurs, FGF21 is related to the NST response in humans. 

Considering exercise in cold environments, plasma and serum FGF21 levels were unchanged after 60 min of cycling at 60% HR_max_ (15–19 °C) and the Yukon Arctic Ultra (−25–−2 °C), respectively [40,43]. Compared with low intensity, high-intensity exercise was suggested to raise the protein expression level of FGF21 in skeletal muscle [52]. Therefore, insufficient exercise intensity in these studies might be the reason for the unchanged FGF21 level. Kozłowska-Flis et al. reported that HIIT alone (90% HR_max_) caused a significant increase in serum FGF21 concentration, whereas in combination with WBC (−110 °C, 3 min), it abolished this effect [44] (Table 2).

The main tissue source of FGF21 after exercise is still unknown. It was suggested that exercise is a key stimulus in inducing the hepatic, but not skeletal muscle, release of FGF21 into the systemic circulation [53]. Different tissue sources of FGF21 in response to exercise and cold exposure might make it difficult to predict FGF21 levels while exercising in the cold. Nonetheless, although no changes in FGF21 were observed after WBC (−110 °C, 3 min) following the HIIT (90% HR_max_), a reduction in glucose levels was shown [44]. The evidence also suggested that the use of physical activity combined with cold exposure can be used as a preventive strategy for some diseases. 

### 3.3. IL-6

As the classic and best-characterized myokine, significant amounts of IL-6 were proved to be released from contracting skeletal muscle during prolonged exercise [31]. Exercise duration, intensity and muscle mass all have effects on the circulating IL-6 level. IL-6 is best known as a pro-inflammatory factor that induces insulin resistance in obese and type-2 diabetic patients [54]. Indeed, IL-6 has dual roles in regulating insulin sensitivity. According to recent reports, the acute increase of muscle-induced IL-6, unlike the chronically elevated IL-6, improves muscle insulin sensitivity [7]. IL-6 was also reported to have beneficial effects on glucose uptake and fatty acid oxidation by activating AMP-activated protein kinase (AMPK) and BAT [17,55,56]. 

Although, plasma concentrations of IL-6, which did not increase with cold exposure, significantly lower WAT UCP1 protein content in IL-6 KO mice, indicating the important role of IL-6 in regulating cold-induced UCP1 expression [57,58], thus mediating WAT browning. Yildirim et al. also demonstrated that cold exposure (10 °C) induced increases in IL-6 mRNA levels in rat brain, liver, lung and heart tissues [59]. In human clinical trials, however, contradictory results were reported with cold exposure [60,61]. The aforementioned exercise- and cold-induced findings suggested that IL-6 might have different regulatory roles during cold exposure and exercise.

As a blood inflammatory marker, IL-6 was mainly investigated with cryotherapy in combination with exercise. Ziemann et al. reported that IL-6 levels increased after a 5-day WBC (−120 °C, twice a day) in combination with a moderate-intensity training program for professional tennis players [62]. However, a significant drop in IL-6 was observed after a single session of WBC (−110 °C) following a volleyball training program [63]. Furthermore, four weeks of resistance training combined with WBC did not change the IL-6 level [45] (Table 2). Therefore, it was speculated that IL-6 concentration could be affected by exercise intensity and duration, and the individual’s endurance capacity. Cold exposure may have a limited influence on IL-6 secretion. The ambiguous changes warrant further investigations.

### 3.4. Metrnl

As an exercise- and cold-induced circulating factor in skeletal muscle and adipose tissue, respectively, Metrnl was first identified in 2014 [32]. Unlike irisin and FGF21, Metrnl induces immune cytokines (IL-4 and IL-13) to induce the expression of UCP1 and other adipose thermogenic genes, thus increasing whole-body energy expenditure. In 2018, Bae found that exercise-induced Metrnl effectively reduces fat accumulation through an obesity mouse model [64]. It was also reported that Metrnl controls insulin sensitivity through the PPARγ pathway in a previous study [65]. This evidence indicates that upregulation of Metrnl may bring benefits to whole-body metabolism, and Metrnl may become a therapeutic target for chronic obesity.

In rodents, Metrnl expression was affected by exercise type and muscle position. A 2-fold increase in circulating Metrnl levels was found post-eccentric exercise. However, in the same study, an endurance exercise training program did not change the Metrnl level [32]. On the other hand, in humans, an acute bout of concurrent exercise increased Metrnl mRNA expression. As for cold exposure and Metrnl expression, cold-induced (4 °C) thermogenic responses were attenuated upon blocking Metrnl actions in vivo, suggesting that Metrnl plays a role in adaptation to cold temperatures [32]. 

When combining exercise with cold exposure, the Metrnl level remained unchanged after the Yukon Arctic Ultra at −25–−2 °C and even declined in overweight women after interval training for 40 min at 16.5–17.5 °C [30,59] (Table 2). The differences in the temperatures adopted were speculated as the reason for the inconsistent results. To the best of our knowledge, the scope of Metrnl function has not yet been determined, and the effects of cold on Metrnl expression in humans warrant further research.

### 3.5. Myostatin

Myostatin was the first identified myokine in 1997, which has been well-known for inhibiting skeletal muscle cell growth and differentiation [33]. Myostatin is a negative regulator of skeletal muscle, and studies have mainly focused on its role in sarcopenia, a progressive skeletal muscle disorder, as well as metabolic diseases. 

Both endurance and resistance training lead to a decrease in myostatin levels [34]. In addition to regulating muscle cell growth, an increase in myostatin was reported to suppress irisin, thus affecting WAT browning and systemic insulin sensitivity [66,67]. Further, Kong et al. recently reported that cold exposure could inhibit myostatin secretion from BAT due to the upregulation of interferon regulatory factor 4 (IRF4) in BATI4KO mice, affirming the role of myostatin in BAT–muscle crosstalk [35]. In humans, concentrations of myostatin were observed to decrease in response to chronic WBC at −110 °C, for a total of 10 sessions completed over two weeks. The decrease was more pronounced in the middle-aged group than in the young group [68]. These results suggest that myostatin expression is affected by low temperature.

Limited studies have investigated changes in myostatin in response to exercise coupled with cold exposure. Jaworska et al. first reported that WBC (−110 °C, 3 times/week) following a 4-week resistance training program is effective in lowering circulating levels of myostatin [45]. However, no significant shifts in myostatin levels were reported after a 2-week volleyball training program combined with WBC (−110 °C, 5 times/week) [63] (Table 2). Therefore, myostatin secretion following resistance training and cold exposure might depend on the presence of a specific exercise stimulus.

**Table 2:** Human studies examining myokine secretion following exercise and cold exposure.

Study (Year)	Population		Exercise Protocol			Temperature	Results
	Sample Size	Mean Age	Type (Duration)	Intensity	Period (Frequency)		
Ulupinar et al. (2021) [39]	27	21 y	Running (40 min)	70% HR_max_	/	0 °C, 12 °C, 24 °C	-0 °C: irisin↑ -ND in adropin
Ozbay et al. (2020) [42]	32	>18 y	Running (40 min)	65–70% HR_max_	18 wk (4 d/wk)	Outdoor: −5–5 °C, Indoor: 21–25 °C	-Outdoor: ND in irisin, HDL-C↑ -Indoor: irisin↓, ND in adropin
Tsuchiya and Goto (2021) [43]	7	23 y	Cycling (60 min)	60% HR_max_	/	Cold: 15–19 °C, Moderate: 24 °C, Hot: 34 °C	-Cold: ND in irisin and FGF21 -Hot: FGF21↑, myostatin↓
Bubak et al. (2017) [41]	12	25 y	Cycling (60 min)	60% W_max_	/	7 °C, 20 °C, 33 °C	-ND in FNDC5 and irisin among the 3 temperatures
Vosselman et al. (2015) [60]	24	Trained:25 y; Sedentary: 23 y	/	/	/	Cool down until shivering occurred	-Trained: FNDC5↑ -ND in irisin and IL-6 between the groups
Coker et al. (2017) [40]	8	44 y	Running (several days)	/	/	−25–−2 °C	-Irisin↑ -ND in FGF21 and Metrnl
Saghebjoo et al. (2018) [69]	13	25 y	Interval training (40 min)	65% HR_max_	/	Warm: 36.5–7.5 °C, Temperate: 24–25 °C, Cold: 16.5–17.5 °C	-Warm: Metrnl↑, IL-4↑ -Temperate: Metrnl↑ -Cold: Metrnl↓
Jaworska et al. (2018) [63]	20	University students	Aerobic + Resistance (>60 min)	/	-Exercise: 2 wk (once a day) -WBC: 2 wk (5 times/wk)	−110 °C	-IGF1↑ -BDNF↑ -IL-6↓ -ND in Myostatin
Kozłowska-Flis et al. (2021) [44]	65	Training (TR): 42 y vs. Training with cryotherapy (TR-WBC): 45 y	HIIT (60 min)	90% HR_max_	-HIIT: 2 wk (3 times/wk)-WBC: 2 wk (10 times in 2 wk)	−110 °C	-TR: FGF21↑, adiponectin↑, ND in irisin
Jaworska et al. (2020) [45]	25	20 y, Cryostimulation (CRY) vs. Control (CON)	Resistance (50 min)	70–80% 1RM	4 wk (3 times/wk)	−110 °C	-CRY: Myostatin↓, IL-15↑ -ND in irisin, IL-6 and BDNF

Abbreviations: ND: no difference; HDL-C: high-density lipoprotein cholesterol; FGF21: fibroblast growth factor 21; mRNA: messenger RNA; FNDC5: fibronectin type III domain containing 5; BAT: brown adipose tissue; IL-6: interleukin 6; Metrnl: Meteorin-like; IL-4: interleukin 4; BDNF: brain-derived neurotrophic factor; IL-15: interleukin 15; HIIT: high-intensity interval training.

## 4. Potential Circulating Factors Related to Exercise and Cold Exposure

**Figure 2:**
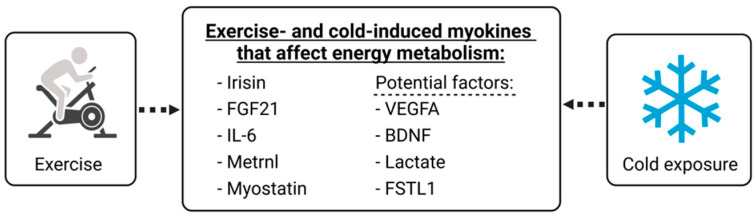
Exercise and cold exposure have been reported to increase energy metabolism by regulating myokines (irisin, FGF21, IL-6, Metrnl and myostatin). In addition, potential myokines (VEGFA, BDNF, Lactate and FSTL1) have shown possibilities in regulating lipid metabolism in response to exercise and/or cold exposure in mice.

Apart from the myokines mentioned above, some other circulating factors might be related to exercise and low temperatures, such as vascular endothelial growth factor A (VEGFA), brain-derived neurotrophic factor (BDNF), follistatin-like protein-1 (FSTL1) and lactate. These factors might also be potential therapeutic targets for metabolic diseases (Figure 2). However, to the best of our knowledge, the effects of exercise in conjunction with cold exposure on these factors have not yet been investigated. Therefore, changes in the factors in response to exercise and cold exposure will be discussed separately.

### 4.1. VEGFA

As an angiogenic factor, vascular endothelial growth factor A (VEGFA) is a major regulator of vascular endothelial cell activation, proliferation and migration. Various studies have shown that physical exercise upregulates VEGFA in mice and humans [70,71,72]. With regard to cold exposure, a doxycycline (Dox)-inducible, BAT-specific VEGFA transgenic overexpression model showed that the expression of both UCP1 and PGC-1α in BAT was upregulated by VEGFA expression during chronic cold exposure. As a result, thermogenesis was increased [73].

### 4.2. BDNF

Although mainly released from the brain, known as a factor improving cognitive function, BDNF was also identified as a contraction-regulated myokine capable of enhancing AMPK activation and, hence, lipid oxidation [74]. It has been previously reported that serum BDNF levels increased after exercise tests and exerted weight-reducing effects in humans and mice [74,75,76]. Besides skeletal muscle, irisin is also expressed in several regions of the brain [77]. Recent studies suggested that FNDC5/irisin could induce BDNF expression, thus serving as a link between the benefits of exercise and reward-related learning [78,79]. Due to the temperature-dependent characteristic of irisin, BDNF secretion is therefore speculated to be involved in cold exposure, too.

### 4.3. Lactate

As the well-known end product of anaerobic glycolysis, lactate levels can rise 20-fold during intense exercise. In addition to being an energy substrate, lactate also plays a role as a signaling molecule, delivering oxidative and gluconeogenic substrates [80,81]. Interestingly, Carrière et al. recently reported an upregulation in circulating lactate levels in response to cold exposure in human cells in vivo, which induced an increase in thermogenic gene expression (Ucp1, Cidea, Fgf21 and Hoxc9) [82]. Therefore, an increase in lactate could contribute to WAT browning.

### 4.4. FSTL1

Follistatin-like protein-1 (FSTL1) is an extracellular glycoprotein from the follistatin family, secreted by adipose, lung, heart and also primary human skeletal muscle cells [56,83]. It was found that FSTL1 was associated with glucose metabolism and insulin resistance by investigating obese individuals [84], suggesting its potential role in energy metabolism. Serum FSTL1 levels significantly increased after a 45-min treadmill test (60% VO_2max_), as well as acute sprint interval training with high intensity [85,86]. Furthermore, recent studies demonstrated that cold stimulation increased FSTL1 expression in Fstl1^+/−^ mice and 3T3-L1 cells but not in young and healthy humans [85,87]. The discrepancies between results need to be further investigated.

## 5. Conclusions and Future Perspectives

According to recent findings, it seems that environmental temperature during exercise can be used to increase adipose tissue metabolism by regulating myokine profiles. Elements of both exercise and environmental temperature need to be considered to develop an optimal exercise regimen.

As a skeletal muscle contraction-induced myokine from skeletal muscle, irisin expression is upregulated when ST occurs in the cold. Key effects exist in exercise intensity and duration as well as temperature and cold exposure time. Unlike irisin, FGF21 is related to NST responses when exposed to prolonged cold. Exercise intensity also affects its expression. Mechanisms underlying the effect of cold exposure on IL-6, Metrnl and myostatin have not yet been investigated but animal models have demonstrated the possibility of increasing energy expenditure via regulating these myokines in the cold. Considering the contradictory results, data from animals should be transferred cautiously to humans. 

To date, there is not sufficient research on myokine responses to cold exposure, especially in humans. Future research needs to be conducted to clarify the mechanisms behind WAT browning induced by myokines. In addition, the effects of gender, age and human body composition (adiposity) on myokine concentration with cold exposure solely or combined with exercise warrant future investigation. Blood collection time, measuring method and intervention duration should be considered rigorously to improve the research quality. Further, although there are potent similarities between exercise and cold exposure, dissimilarities exist. Cold exposure triggers mechanisms in the human body to compensate for heat loss, while exercise increases heat production. Therefore, when the two stimuli are combined, the physiological responses become more complex. Whether antagonism exists between the two stimuli and which organ plays the main role in releasing the secretory factors remain to be investigated. To solidify the findings in humans, well-designed and controlled clinical studies are needed.

## Figures and Tables

**Table 1:** Myokine changes in response to exercise and cold exposure.

Myokine	Related to Exercise		Related to Cold Exposure	
	Rodent	Human	Rodent	Human
Irisin	Yes [26]	Yes [26]	Not sure	Yes [27]
FGF21	Yes [28]	Not sure	Yes [29]	Yes [27]
IL-6	Yes [30]	Yes [31]	Not sure	Not sure
Metrnl	Yes [32]	Yes [32]	Yes [32]	Not sure
Myostatin	Yes [33]	Yes [34]	Yes [35]	Not sure
